# Presolar Isotopic Signatures in Meteorites and Comets: New Insights from the Rosetta Mission to Comet 67P/Churyumov–Gerasimenko

**DOI:** 10.1007/s11214-018-0540-3

**Published:** 2018-09-06

**Authors:** Peter Hoppe, Martin Rubin, Kathrin Altwegg

**Affiliations:** 1 Max Planck Institute for Chemistry, Hahn-Meitner-Weg 1, 55128 Mainz, Germany; 2 Physikalisches Institut, University of Bern, Sidlerstrasse 5, 3012 Bern, Switzerland; 3 Center for Space and Habitability, University of Bern, Sidlerstrasse 5, 3012 Bern, Switzerland

**Keywords:** Comets, Meteorites, Isotopic compositions, Rosetta mission

## Abstract

Comets are considered the most primitive planetary bodies in our Solar System, i.e., they should have best preserved the solid components of the matter from which our Solar System formed. ESA’s recent Rosetta mission to Jupiter family comet 67P/Churyumov–Gerasimenko (67P/CG) has provided a wealth of isotope data which expanded the existing data sets on isotopic compositions of comets considerably. In this paper we review our current knowledge on the isotopic compositions of H, C, N, O, Si, S, Ar, and Xe in primitive Solar System materials studied in terrestrial laboratories and how the Rosetta data acquired with the ROSINA (Rosetta Orbiter Sensor for Ion and Neutral Analysis) and COSIMA (COmetary Secondary Ion Mass Analyzer) mass spectrometer fit into this picture. The H, Si, S, and Xe isotope data of comet 67P/CG suggest that this comet might be particularly primitive and might have preserved large amounts of unprocessed presolar matter. We address the question whether the refractory Si component of 67P/CG contains a presolar isotopic fingerprint from a nearby Type II supernova (SN) and discuss to which extent C and O isotope anomalies originating from presolar grains should be observable in dust from 67P/CG. Finally, we explore whether the isotopic fingerprint of a potential late SN contribution to the formation site of 67P/CG in the solar nebula can be seen in the volatile component of 67P/CG.

## Introduction

Our Solar System formed from the collapse of an interstellar gas and dust cloud some 4.6 billion years ago. In this event, much of the solid matter that went into the making of the Solar System was destroyed or severely altered by heating due to release of gravitational energy. However, some of the interstellar dust grains, ices, and organics survived this event largely unaltered. This pristine matter, together with thermally processed interstellar matter, and newly condensed minerals from the solar nebula formed an accretion disk around the young Sun from which comets, asteroids, and the planets later formed (e.g., Dauphas and Chaussidon 2011). Thermal metamorphism on rocky planets finally led to the complete destruction of unaltered interstellar matter in those objects; in comets and small asteroids, however, thermal (and aqueous) alteration was less severe which provided favorable conditions for the survival of pristine interstellar matter.

Interstellar dust consists of stardust and dust that forms in the interstellar medium (ISM). From astronomical observations, it is well known that dust forms in dense and hot stellar winds and in the ejecta of stellar explosions. In contrast, it is currently not known how dust may form at the cool and low-density conditions typically prevailing in the ISM. However, a comparison between the inferred rates of stellar dust production and its destruction in the ISM led to the conclusion that there must be a source of dust formation in the ISM (e.g., Zhukovska et al. 2008). In a recent work, Hoppe et al. (2017) estimated that a few percent of the dust contained in the interstellar gas and dust cloud from which our Solar System formed was stardust, in line with predictions from recent theoretical models for the composition of interstellar dust (Zhukovska et al. 2016). Stardust can be identified by large isotope abundance anomalies relative to solar isotope abundances, the fingerprints of nucleosynthetic processes in their parent stars (Zinner 2014). In contrast, dust that formed in the interstellar cloud predating our Solar System, e.g., silicates, must not necessarily have isotope abundance anomalies. Organics and ices with an interstellar or outer protoplanetary disk origin, on the other hand, may carry large isotope anomalies in H and N (Busemann et al. 2006, and references therein), which can be produced by ion-molecule reactions.

Presolar isotopic signatures have been identified in recent years in a variety of primitive Solar System materials, namely, in undifferentiated meteorites (chondrites), which are fragments of asteroids, interplanetary dust particles (IDPs) collected in the stratosphere, ultra-carbonaceous Antarctic micrometeorites (UCAMMs), and comets. The most comprehensive information on presolar material is available for chondrites, since these samples can be studied in terrestrial laboratories in great detail and are available in large quantities. Some IDPs, in particular anhydrous chondritic porous IDPs (CP-IDPs) (Ishii et al. 2008), and UCAMMs (Duprat et al. 2010) may have a cometary origin. As IDPs and UCAMMs are only several micrometers in size, much less material is available for laboratory studies than for chondrites. Matter from comet 81P/Wild 2, returned to Earth in 2006 by NASA’s Stardust mission, complements the reservoir of cometary matter available for studies in terrestrial laboratories, although only about 1 mg of mass was collected (Brownlee et al. 2006). In-situ measurements by the Giotto spacecraft on Oort cloud comet 1P/Halley (Balsiger et al. 1995; Eberhardt et al. 1995; Jessberger and Kissel 1991) provided isotope data for cometary gas and dust. Additional isotope data were acquired by ground-based spectroscopic observations for a variety of comets (Bockelée-Morvan et al. 2015).

Stardust, also known as “presolar grains”, has been found in all kinds of primitive Solar System materials available for laboratory studies (Zinner 2014). Among the identified stardust minerals are silicon carbide (SiC), graphite, silicon nitride (Si_3_N_4_), refractory oxides, such as spinel (MgAl_2_O_4_), corundum and other forms of Al_2_O_3_, and various silicates. Also, meteoritic nanodiamonds have been considered to have a presolar origin. However, a stellar origin is questionable and these grains might have formed by shock in the ISM or even in the Solar System. Among the presolar grains with a stellar origin, silicates are most abundant. Certain types of presolar grains, namely, carbonaceous grains, Si_3_N_4_, and, with restrictions, also refractory oxides can be separated from meteorites by chemical treatments. This does not hold for presolar silicates and that is why they remained unrecognized for a long time. Only the application of high-resolution ion imaging techniques with the NanoSIMS ion probe (Hoppe et al. 2013) made their discovery possible at the beginning of this millennium (Messenger et al. 2003). Presolar grains exhibit large isotopic anomalies in their major, minor, and trace elements with isotopic compositions sometimes deviating by orders of magnitude from those of Solar System materials (Zinner 2014). Based on a comparison with stellar models and astronomical observations it is now well established that the majority of meteoritic stardust grains formed in the winds of low-mass (1–3 M_⊙_) asymptotic giant branch (AGB) stars; important contributions also come from Type II supernovae (SNe) and a small fraction of presolar grains appear to originate from novae. Besides presolar grains, organics with large H and N isotope anomalies have been found in chondrites, IDPs, and UCAMMs (Busemann et al. 2006; Duprat et al. 2010; Messenger 2000). Large enrichments in ^17^O and ^18^O in meteoritic cosmic symplectite (so-called COS phase) have been interpreted to represent the O-isotopic signature of primordial water in the solar nebula (Sakamoto et al. 2007).

Comets are considered the most primitive planetary bodies in our Solar System, i.e., they should have best preserved the solid components of the matter from which our Solar System formed (Bockelée-Morvan et al. 2015; Engrand et al. 2016), despite the fact that studies of matter from comet 81P/Wild 2 have shown that the non-volatile portion of this comet contains significant amounts of materials that have a Solar System origin (Brownlee et al. 2006). ESA’s recent Rosetta mission to Jupiter family comet (JFC) 67P/Churyumov–Gerasimenko (67P/CG) has provided a wealth of isotope data which expanded the existing data sets on isotopic compositions in comets considerably and which will be the subject of this paper. Prior to Rosetta various presolar signatures were found in cometary matter: (i) Identification of C-rich particles with high ^12^C/^13^C from comet 1P/Halley (Jessberger and Kissel 1991). (ii) Fingerprints of interstellar (or outer protoplanetary disk) chemistry indicated by large D and ^15^N enrichments as well as presence of C- and O-rich presolar grains in matter from comet 81P/Wild 2 (McKeegan et al. 2006). These presolar grains represent some 600–1100 ppm of the cometary matter (Floss et al. 2013; Leitner et al. 2012), compared to about 200 ppm in chondrites (Floss and Haenecour 2016). (iii) CP-IDPs, especially those associated with comet 26P/Grigg–Skjellerup (26P/GS), show high abundances of presolar grains, a few 100 ppm on average and up to 1.5% in one particle (Busemann et al. 2009), which is compatible with the estimate on the stardust abundance in the interstellar gas and dust cloud from which our Solar System formed (Hoppe et al. 2017). (iv) Remote spectroscopic observations of comets indicate variable D enrichments in water and organics as well as variable ^15^N enrichments in organic molecules (Bockelée-Morvan et al. 2015). Altogether, studies of cometary matter have revealed the wide-spread presence of presolar signatures, but apparently at different abundance levels.

Here, we will focus on isotope data recently obtained by Rosetta for comet 67P/CG. Isotope measurements were performed by the ROSINA (Rosetta Orbiter Sensor for Ion and Neutral Analysis) mass spectrometer (Balsiger et al. 2007) for the volatile component in the cometary coma and for the refractory component of 67P/CG sputtered by solar wind ions; as of June 2018, isotope data are available for H, C, O, Si, S, Ar, and Xe (Altwegg et al. 2015, 2017; Balsiger et al. 2015; Calmonte et al. 2017; Hässig et al. 2017; Marty et al. 2017; Rubin et al. 2017). Dust particles from the inner coma were studied by COSIMA (COmetary Secondary Ion Mass Analyzer) (Hilchenbach et al. 2016); as of June 2018 isotope data for dust have been reported for O and S only (Paquette et al. 2017; Paquette et al. 2018). Unfortunately, the lander Philae bounced off the comet’s surface several times and ended up in shadow, which significantly limited available power and measurement time during the initial science phase. Also, attempts on a stable communication later during the mission failed and therefore no isotope data from the lander is available from this part of Rosetta.

In this paper, we will briefly review our current knowledge on the isotopic compositions of H, C, N, O, Si, S, Ar, and Xe in primitive Solar System materials studied in terrestrial laboratories and how the Rosetta data (ROSINA data as of June 2018, complemented by COSIMA isotope data for O and S) fit into this picture. As H isotope measurements on water in 67P/CG revealed the largest D enrichments found in cometary water to date (Altwegg et al. 2015), besides those reported for Ort cloud comet Lemmon (Biver et al. 2016), 67P/CG is a promising target to search for presolar components known from studies of chondrites and IDPs. We will address the question whether the refractory Si component of 67P/CG contains a presolar isotopic fingerprint and will discuss to which extent C and O isotope anomalies originating from presolar grains should be observable in dust from 67P/CG. Finally, we will deal with the question whether the isotope fingerprint of a potential late Type II supernova (SN) contribution to the formation site of 67P/CG in the solar nebula can be seen in the volatile component of this comet.

## Isotopic Compositions: Comet 67P/CG in the Context of Other Primitive Solar System Materials

**Fig. 1:**
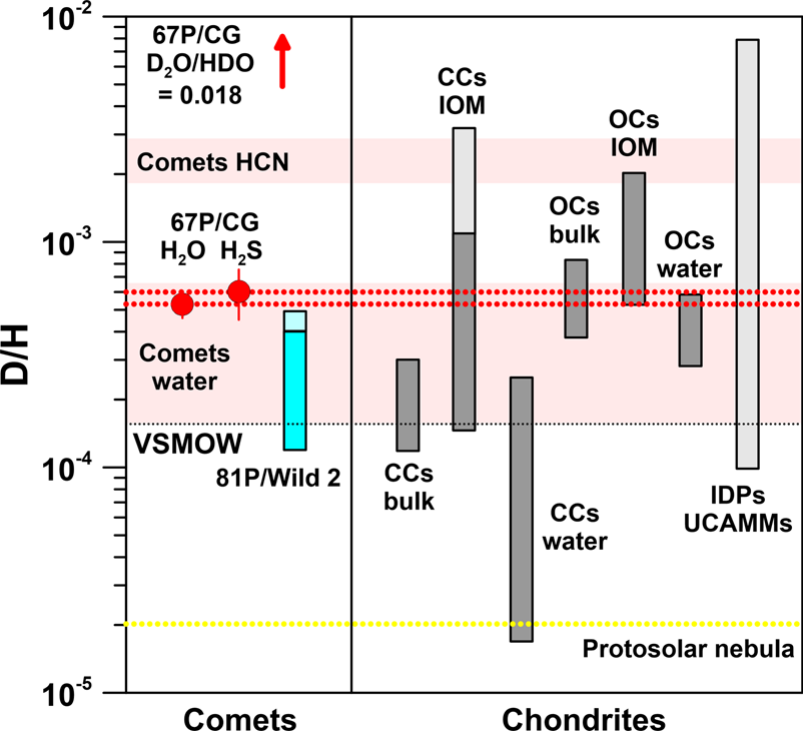
Hydrogen-isotopic compositions in various Solar System objects. CCs: carbonaceous chondrites; OCs: ordinary chondrites; IOM: insoluble organic matter; VSMOW: Vienna standard mean ocean water. The light-blue and light-grey shaded extensions in the bars for comet 81P/Wild 2, CCs IOM, and IDPs and UCAMMs indicate D/H ranges observed in micrometer-sized hotspots. The pink-shaded horizontal bars represent D/H ratios of water and HCN in 8 comets. The value of D_2_O/HDO in 67P/CG is off-scale, as indicated by the arrow. Data sources: protosolar nebula: Geiss and Gloeckler (2003); 67P/CG: Altwegg et al. (2015) (D/H derived from HDO/H_2_O); Altwegg et al. (2017) (D/H derived from HDS/H_2_S); chondrites: Alexander et al. (2007, 2010, 2012); Busemann et al. (2006), Kerridge (1985), Pearson et al. (2001), Yang and Epstein (1983); IDPs and UCAMMs: Duprat et al. (2010), Messenger (2000); 81P/Wild 2: McKeegan et al. (2006); comets water and HCN: Biver et al. (2016), Bockelée-Morvan et al. (2015). Errors are 1$\sigma$

**Fig. 2:**
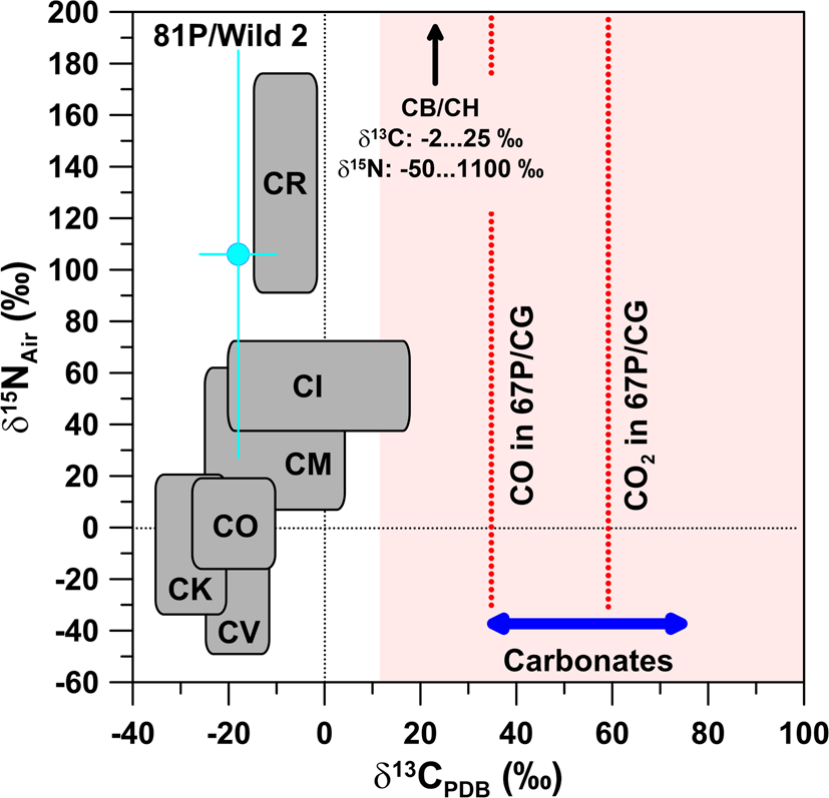
Carbon- and N-isotopic compositions, given in permil deviation from terrestrial PDB and air standards, respectively, of bulk carbonaceous chondrites, carbonates from chondrites (C data only), comets 81P/Wild 2, and 67P/CG (C data only). The N-isotopic compositions of CB/CH chondrites are off-scale. Carbon- and N-isotopic compositions of 81P/Wild 2 represent bulk compositions inferred from residues in impact craters on Al foils from NASA’s Stardust mission; errors are 1$\sigma$. The blue arrow represents the range of $\delta ^{13}$C values of carbonates. The data for 67P/CG (red dotted lines) are for CO_2_ and CO. The pink colored area represents the 1$\sigma$ uncertainty for $\delta ^{13}$C in CO_2_; the 1$\sigma$ uncertainty for $\delta ^{13}$C in CO extends over the whole plot range and is not shown. Data sources: 67P/CG: Hässig et al. (2017); bulk chondrites: Alexander et al. (2012), Franchi et al. (1986), Grady and Pillinger (1990), Ivanova et al. (2008), Kerridge (1985), Pearson et al. (2006), Sugiura et al. (2000); carbonates: Fujiya et al. (2015); 81P/Wild 2: Stadermann et al. (2008)

**Fig. 3:**
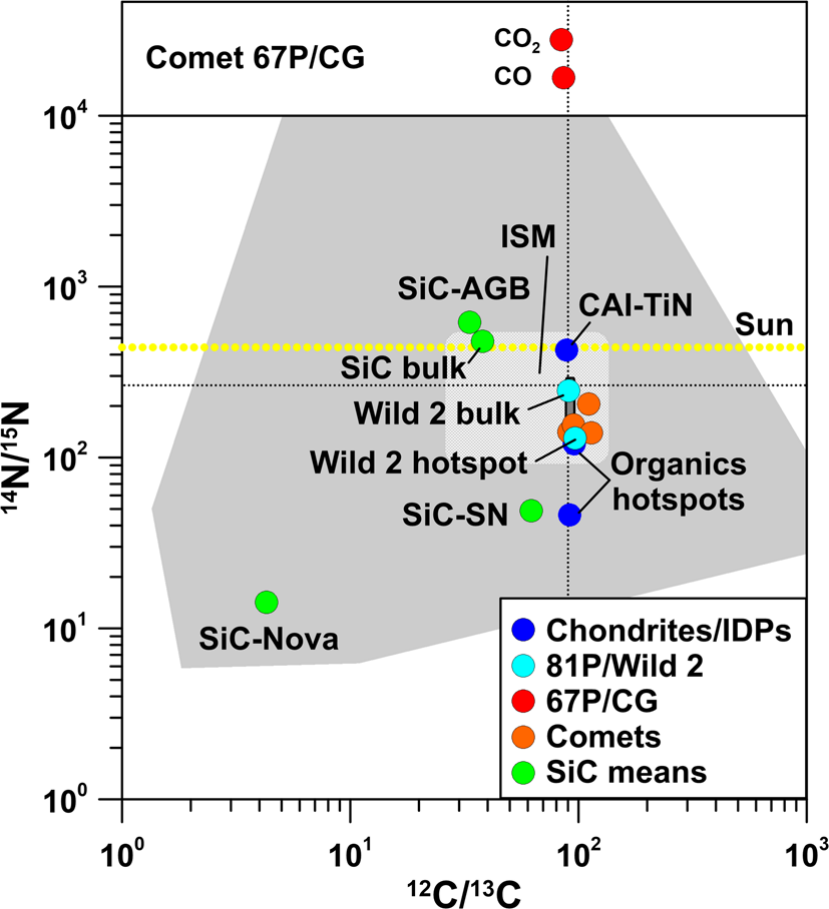
Carbon- and N-isotopic ratios of specific components in chondrites and IDPs, comets 81P/Wild 2 and 67P/CG (C data only, upper panel), and other comets in comparison to presolar SiC grains (mean values for grains from AGB stars, supernovae, and novae, and for bulk SiC) and the ISM. Carbon- and N-isotopic compositions for Wild 2 were inferred from residues in impact craters on Al foils from NASA’s Stardust mission. The medium-grey area represents the data of individual SiC grains, the light-grey hatched area those of the ISM, and the dark-grey area those for the bulk compositions of carbonaceous chondrites. The black dotted lines indicate the terrestrial PDB (C) and air (N) values. Data sources: chondrites and IDPs: Briani et al. (2009), Floss et al. (2004), Meibom et al. (2007); 81P/Wild 2: Stadermann et al. (2008); 67P/CG: Hässig et al. (2017) (derived from CO_2_); Rubin et al. (2017) (derived from CO); other comets: Bockelée-Morvan et al. (2015); Sun: Marty et al. (2011); presolar SiC: Amari et al. (2001a, 2001b), Besmehn and Hoppe (2003); Hoppe et al. (1994, 1996, 2000, 2010); Huss et al. (1997), Lin et al. (2002), Liu et al. (2016), Marhas et al. (2008), Nittler (1996), Nittler and Hoppe (2005), Xu et al. (2015); ISM: Furi and Marty (2015), Wilson (1999)

In this chapter, we present and discuss the isotopic compositions of H, C, N (no data yet for 67P/CG), O, Si, S, Ar, and Xe in various primitive Solar System materials. The isotopic compositions of distinct components in primitive meteorites/IDPs/UCAMMs, comets (81P/Wild 2, 67P/CG, and others), the Sun, the protosolar nebula, the ISM, and protostar regions are listed in Table 1 and/or are displayed in graphical form in Figs. 1, 2, 3, 5, 6, 8, 10, and 12. Terrestrial reference values for isotopic compositions are given in Table 2.

**Table 1:** Isotopic compositions of distinct components in primitive meteorites, IDPs/UCAMMs, comets 81P/Wild 2 and 67P/CG, the ISM, and protostar regions

Element	Component^1^	Isotopic composition^2^^,^^3^		References
H		$\delta\, \mathrm{D}_{\mathrm{VSMOW}}$ (‰)		
	Protosolar nebula	−870		[1]
	Bulk CCs/OCs	−230… + 4400		[2–6]
	Organics CCs/OCs	Up to +19000		[2,7,8]
	Water CCs/OCs	−895… + 2800		[2,3]
	IDPs/UCCAMs	−400… + 50000		[39,40]
	81P/Wild 2	−240… + 2200		[41]
	Protostar regions	Up to +260000		[52]
	$\mathbf{67P}\boldsymbol{/}\mathbf{CG}\ \mathbf{H}_{\mathbf{2}}\mathbf{O}$	**+2400 ± 225**		**[9]**
	$\mathbf{67P}\boldsymbol{/}\mathbf{CG}\ \mathbf{H}_{\mathbf{2}}\mathbf{S}$	**+2870 ± 970**		**[52]**
C		$\delta ^{13}\mathrm{C}_{\mathrm{PDB}}$ (‰)		
	Bulk CCs	−30… + 25		[3,4,10]
	Sun	−92		[37]
	Organics CCs/IDPs	−70…0		[7,11]
	Carbonates	+35… + 75		[12]
	81P/Wild 2 (bulk)	−18 ± 8		[48]
	Presolar SiC	−1000… + 70 000		[13]
	ISM	−300… + 2600		[53,54]
	Local ISM	+310 ± 290		
	**67P/CG CO**	**+34 ± 103**		**[35]**
	$\mathbf{67P}\boldsymbol{/}\mathbf{CG}\ \mathbf{CO}_{\mathbf{2}}$	**+59 ± 48**		**[36]**
N		$\delta^{15} \mathrm{N}$ (‰)		
	Bulk CCs	−50… + 1100		[3, 4, 10, 14–17]
	Sun	−383		[18]
	CAIs	−360		[42]
	Organics CCs/IDPs	−50… + 4900		[7,8,19]
	81P/Wild 2	Up to +1100		[48]
	Presolar SiC	−1000… + 39 000		[13]
	ISM	−500… + 2000		[55]
O		$\delta ^{17}\mathrm{O}_{\mathrm{VSMOW}}\ (\permil)$	$\delta ^{18}\mathrm{O}_{\mathrm{VSMOW}}$ (‰)	
	Bulk CCs	−6… + 9	−2… + 16	[20,21]
	Sun	−59	−59	[22]
	CAIs, hibonites	−55…0	−55…0	[43–46, 56, 57]
	COS (water)	+180	+180	[23]
	81P/Wild 2 (bulk)	−11 ± 18	−11 ± 16	[48]
	Presolar Ox./Sil.	−900… + 120 000	−1000…13 000	[13]
	ISM		−170… + 1500	[53]
	$\mathbf{67P}\boldsymbol{/}\mathbf{CG}\ \mathbf{CO}_{\mathbf{2}}$		**+10 ± 16**	**[36]**
	**67P/CG** **Dust**		**−2 ± 60**	**[58]**
Si		$\delta ^{29} \mathrm{Si}$ (‰)	$\delta ^{30} \mathrm{Si}$ (‰)	
	Bulk CCs/OCs	∼0	∼0	[24]
	FUN inclusion	13	23	[47]
	Presolar SiC	−700… + 2500	−800… + 2900	[13]
	**67P/CG**	**−145 ± 98**	**−214 ± 115**	**[35]**
S		$\delta ^{33}$S (‰)	$\delta ^{34}$S (‰)	
	Bulk CCs/OCs	−1… + 3	−3… + 6	[25–27]
	81P/Wild 2 (bulk)	−17 ± 37	−1 ± 18	[49]
	Presolar SiC	−940… ∼ 0	−940… ∼ 0	[28–32]
	**67P/CG** **H**$_{\mathbf{2}}$**S** (**10/14**)	**−321 ± 32**	**−40 ± 19**	**[33]**
	**67P/CG** **CS**$_{\mathbf{2}}$ (**10/14**)	**−192 ± 35**	**−69 ± 24**	**[33]**
	**67P/CG** **CS**$_{\textbf{2}}$ (**05/16**)	**−161 ± 42**	**−108 ± 23**	**[33]**
	**67P/CG** **OCS** (**10/14**)		**−99 ± 66**	**[33]**
	**67P/CG** **OCS** (**05/16**)	**−230 ± 58**	**−5 ± 14**	**[33]**
	**67P/CG** **Mean** **Volat.**	**−302 ± 29**	**−41 ± 17**	**[33]**
	**67P/CG** **Mean** **Dust**		**+48 ± 129**	**[51]**
Ar		Isotopic signature		
	Q	∼ normal (SW)		[34]
	P3 (diamond)	∼ normal (SW)		[34]
	HL (diamond)	depleted in ^36^Ar		[34]
	G (SiC)	depleted in ^36^Ar		[34]
	N (SiC)	∼ normal (SW)		[34]
	**67P/CG**	$^{\mathbf{36}}\mathbf{Ar}\boldsymbol{/}^{\mathbf{38}}\mathbf{Ar} \boldsymbol{=} \mathbf{5.4} \boldsymbol{\pm} \mathbf{1.4}$		**[50]**
Xe		Isotopic signature		
	Q	∼ normal (SW)		[34]
	P3 (diamond)	∼ normal (SW)		[34]
	HL (diamond)	enriched in ^124,126,134,136^Xe		[34]
	P6 (diamond)	∼ normal (SW)		[34]
	G (SiC)	enr. in ^128,130^Xe, depl. in ^124,126,129,131,134^Xe		[34]
	N (SiC)	∼ normal (SW)		[34]
	**67P/CG**	**enriched in** $^{\mathbf{129}}$**Xe**, **depleted** **in** $^{\mathbf{134,136}}$**Xe**		**[38]**

^1^CCs: Carbonaceous chondrites, OCs: Ordinary chondrites, COS: Cosmic symplectite, Ox/Sil: Oxides/Silicates; ^2^Errors are 1*σ*; ^3^$\delta ^{\mathrm{i}}X =((^{\mathrm{i}}X/{}^{\mathrm{ref}}X)_{\mathrm{sample}}/ ((^{\mathrm{i}} X/^{\mathrm{ref}}X)_{\mathrm{standard}} - 1) \times 1000$; for standard ratios see Table 2 [1] (Geiss and Gloeckler 2003) [2] (Alexander et al. 2010) [3] (Alexander et al. 2012) [4] (Kerridge 1985) [5] (Pearson et al. 2001) [6] (Yang and Epstein 1984) [7] (Alexander et al. 2007) [8] (Busemann et al. 2006) [9] (Altwegg et al. 2015) [10] (Pearson et al. 2006) [11] (Floss et al. 2004) [12] (Fujiya et al. 2015) [13] (Hynes and Gyngard 2009) [14] (Franchi et al. 1986) [15] (Grady and Pillinger 1990) [16] (Ivanova et al. 2008) [17] (Sugiura et al. 2000) [18] (Marty et al. 2011) [19] (Briani et al. 2009) [20] (Clayton 2004) [21] (Lodders and Fegley 1998) [22] (McKeegan et al. 2011) [23] (Sakamoto et al. 2007) [24] (Poitrasson 2017) [25] (Bullock et al. 2010) [26] (Gao and Thiemens 1993b) [27] (Gao and Thiemens 1993a) [28] (Gyngard et al. 2010b) [29] (Hoppe et al. 2012) [30] (Hoppe et al. 2017) [31] (Liu et al. 2016) [32] (Xu et al. 2015) [33] (Calmonte et al. 2017) [34] (Ott 2014) [35] (Rubin et al. 2017) [36] (Hässig et al. 2017) [37] (Hashizume et al. 2004) [38] (Marty et al. 2017) [39] (Messenger 2000) [40] (Duprat et al. 2010) [41] (McKeegan et al. 2006) [42] (Meibom et al. 2007) [43] (Aléon et al. 2002) [44] (Fagan et al. 2002) [45] (Krot et al. 2002) [46] (Liu et al. 2009) [47] (Clayton et al. 1978) [48] (Stadermann et al. 2008) [49] (Heck et al. 2012) [50] (Balsiger et al. 2015) [51] (Paquette et al. 2017) [52] (Altwegg et al. 2017) [53] (Wilson 1999) [54] (Milam et al. 2005) [55] (Furi and Marty 2015) [56] (Kööp et al. 2016a) [57] (Kööp et al. 2016b) [58] (Paquette et al. 2018)

**Table 2:** Terrestrial reference values for H-, C-, N-, O-, Si-, and S-isotopic compositions

Isotope ratio	Reference material	Reference value
D/H	VSMOW	0.00015576
^13^C/^12^C	PDB	0.0112372
^15^N/^14^N	Air	0.0036765
^17^O/^16^O	VSMOW	0.0003799
^18^O/^16^O	VSMOW	0.0020052
^29^Si/^28^Si	NIST NBS 28	0.050804
^30^Si/^28^Si	NIST NBS 28	0.033532
^33^S/^32^S	VCDT	0.0078773
^34^S/^32^S	VCDT	0.0441626

### Hydrogen

D/H ratios of water are available for a large number of comets (Biver et al. 2016; Bockelée-Morvan et al. 2015). The D/H ratios vary between the terrestrial VSMOW value and strong D enrichments of up to a factor of ∼4 in the Oort cloud comet Lemmon (Fig. 1). The D/H ratio of ($5.3 \pm 0.7) \times 10^{-4}$ of water in 67P/CG (derived from HDO/H_2_O with $\mbox{D/H} = 0.5 \times \mbox{HDO/H}_{2}\mathrm{O}$; Altwegg et al. 2015) falls at the upper end of D/H ratios in cometary water and is compatible with the D/H ratio in Lemmon if experimental uncertainties are considered. 67P/CG does not only exhibit a high D/H of water but also has a high D_2_O/HDO (Altwegg et al. 2017) (Fig. 1). The value (D_2_O/HDO)/(HDO/H_2_O) of 17 (statistically one would have expected a value of 1/4) has been explained by dust grain chemistry in the presolar cloud (Furuya et al. 2016) and is in accordance to elevated D/H ratios in water observed in solar-type protostar regions (Coutens et al. 2012, 2013, 2014; van Dishoeck et al. 1995). The D/H ratio of water in the ISM is not well constrained, with $\mbox{HDO/H}_{2}\mathrm{O} < (2\mbox{--}5) \times 10^{-3}$ (Furuya et al. 2016, and references therein). A high D/H ratio of $2.3 \times 10^{-3}$ (i.e., 15× VSMOW) was observed for HCN in comet Hale–Bopp and upper limits determined for other comets are compatible with this (Bockelée-Morvan et al. 2015, and references therein). An elevated D/H ratio of $6 \times 10^{-4}$, again comparable to solar-type protostar regions, was also observed in HDS/H_2_S (Altwegg et al. 2017). Only upper limits were obtained for several other molecules in comets, all of which are compatible with strong D enrichments (Bockelée-Morvan et al. 2015, and references therein). Measured D/H ratios in matter from comet 81P/Wild 2 are not molecule-specific and show D enrichments associated with carbonaceous matter with D/H ratios of up to $5 \times 10^{-4}$ (McKeegan et al. 2006) (Fig. 1).

Brown et al. (2012) and Moores et al. (2012) measured the D/H ratio in water vapor evolved from sublimation of crystalline ice and dusty ice in laboratory experiments and observed that sublimation occurred in bursts, with significant variations in the D/H ratio. This raises the question whether the D/H ratio measured in water vapor is representative of the cometary ice from which it evaporated. However, at least for the measurements on 67P/CG no significant fractionation of D/H between ice and water vapor is expected, for the following reasons: (i) The measured D/H in water remained constant over the whole mission; (ii) the rate of erosion of ice was very large so that no water could have remained behind on the comet, which would have been a prerequisite for D/H fractionation (Altwegg et al. 2017).

The D/H ratios of cometary water and organics are distinctly higher than the D/H ratio of $2.0 \times 10^{-5}$ of H_2_ in the protosolar nebula and of terrestrial water. A similar picture emerges from the H isotope data of CP-IDPs and UCAMMs of putative cometary origins. At the micrometer scale, CP-IDPs show D/H ratios up to $8 \times 10^{-3}$ (50× VSMOW; Messenger 2000) and also UCAMMs show D/H ratios of 10–30× VSMOW, sometimes even at the >10 micrometer scale (Duprat et al. 2010) (Fig. 1). These D enrichments are associated with organic matter.

Water in carbonaceous chondrites (CCs) has D/H ratios in between protosolar H_2_ and terrestrial water (Alexander et al. 2010, 2012) (Fig. 1). Insoluble organic matter (IOM), on the other hand, shows strong D enrichments of up to 7× (at the bulk scale) and, respectively, 20× (at the micrometer scale) the VSMOW value (Alexander et al. 2007, 2010; Busemann et al. 2006) (Fig. 1). In contrast to CCs, water in ordinary chondrites (OCs) shows strong enrichments in D, with a maximum D/H ratio comparable to that of 67P/CG (Alexander et al. 2010, 2012) (Fig. 1). IOM in OCs exhibits extreme D enrichments of up to 13× relative to VSMOW, even at the bulk scale (Alexander et al. 2007, 2010). These D enrichments are not thought to be a signature of H in the presolar cloud, but are most likely caused by loss of isotopically light H_2_ from oxidation of Fe by water and later isotope exchange between water and IOM (Alexander et al. 2010).

The overall picture that emerges from these H isotope data is that organics show generally very large D enrichments in all kinds of primitive Solar System objects, with D/H ratios of up to 50× the VSMOW value. For water the situation is more complex, but if the strong D enrichments of OCs are ignored, which might be a secondary alteration effect, then comets show the largest D enrichments of water. Among the comets, 67P/CG has a D/H at the upper end of observed values, which suggests that this comet might be particularly primitive and might have preserved large amounts of presolar matter, including refractory presolar grains.

### Carbon and Nitrogen

Bulk carbonaceous chondrites have ^12^C/^13^C ratios close to the terrestrial PDB standard (Alexander et al. 2012; Kerridge 1985; Pearson et al. 2006) (Figs. 2 and 3). On a bulk scale, N-isotopic compositions are typically within a few percent of terrestrial air, but certain groups of chondrites (CR, CB/CH) also exhibit strong ^15^N enrichments of more than a factor of 2 (Alexander et al. 2012; Franchi et al. 1986; Grady and Pillinger 1990; Ivanova et al. 2008; Kerridge 1985; Pearson et al. 2006; Sugiura et al. 2000) (Figs. 2 and 3). Even larger ^15^N enrichments are seen for organics in chondrites and IDPs, with maximum ^15^N enrichments of up to a factor of 6 on a micrometer scale, which are sometimes connected to small ^13^C depletions of a few percent (Alexander et al. 2007; Briani et al. 2009; Floss and Stadermann 2004) (Fig. 3). In contrast, the Sun has a ^14^N/^15^N ratio about 40% higher than that of terrestrial air (Marty et al. 2011), i.e., the ^15^N enrichments observed in chondrites and IDPs are even larger relative to this reference. A similar ^14^N/^15^N ratio was determined for the rare mineral osbornite (TiN) in a calcium–aluminum-rich inclusion (CAI), believed to represent the first solids in the Solar System, in the unusual carbonaceous chondrite Isheyevo (Meibom et al. 2007). Observations of volatiles in local and galactic molecular clouds hint at a strong diversity in the ^15^N abundance, reaching from a factor 2 depleted to a factor 2 enhanced with respect to the terrestrial ratio (Furi and Marty 2015).

The general picture of relatively small C-isotopic anomalies and significant ^15^N enrichments also holds for cometary gas. Ground-based spectroscopic measurements of C-isotopic compositions of C_2_, CN, and HCN gave C-isotopic ratios largely compatible (within a few percent) with the terrestrial PDB value, although there is a tendency for small depletions in ^13^C (Bockelée-Morvan et al. 2015, and references therein). In contrast, the N-isotopic compositions of HCN, CN, and NH_2_ show large enrichments in ^15^N of up to a factor of 3 relative to terrestrial air (Bockelée-Morvan et al. 2015, and references therein) (Fig. 3). Similar observations for C- and N-isotopic signatures were made for samples from comet 81P/Wild 2 returned by NASA’s Stardust mission (Stadermann et al. 2008) (Figs. 2 and 3).

For comet 67P/CG C-isotopic data are available for CO and CO_2_ from sublimated ice. CO_2_, believed to be derived from CO by chemical reactions, exhibits slight enrichments in ^13^C of ∼60$\permil$ relative to PDB (Hässig et al. 2017), i.e., in Fig. 2 it plots to the right of bulk carbonaceous chondrites and comet 81P/Wild 2, but overlaps with carbonates in chondrites (Fujiya et al. 2015). With reference to solar wind C, which has $^{12}\mbox{C/}^{13}\mbox{C} = 98 \pm 2$ (Hashizume et al. 2004), the ^13^C enrichment of CO_2_ is 140$\permil$. The small ^13^C enrichment has been taken as evidence for formation of 67P/CG at >25 AU (Hässig et al. 2017). Strong variations in the ^12^C/^13^C ratio from 25 to >100 have been observed in H_2_CO and CO in the ISM, possibly depending on the distance from the Galactic center (Wilson 1999), with a local value of $68 \pm 15$ (Milam et al. 2005). The C-isotopic ratio of CO is compatible with that of CO_2_, albeit within a large experimental uncertainty due to interferences with other peaks and fragmentation of CO_2_ into CO inside the instrument (Rubin et al. 2017). N_2_ has been detected in the coma of 67P/CG (Rubin et al. 2015), but no N isotope data are available yet for 67P/CG. It will be interesting to see whether ^15^N enrichments are present and whether they exceed those observed in other comets and maybe even those of meteoritic organics which would be another piece of evidence that 67P/CG might be a particularly primitive body.

**Fig. 4:**
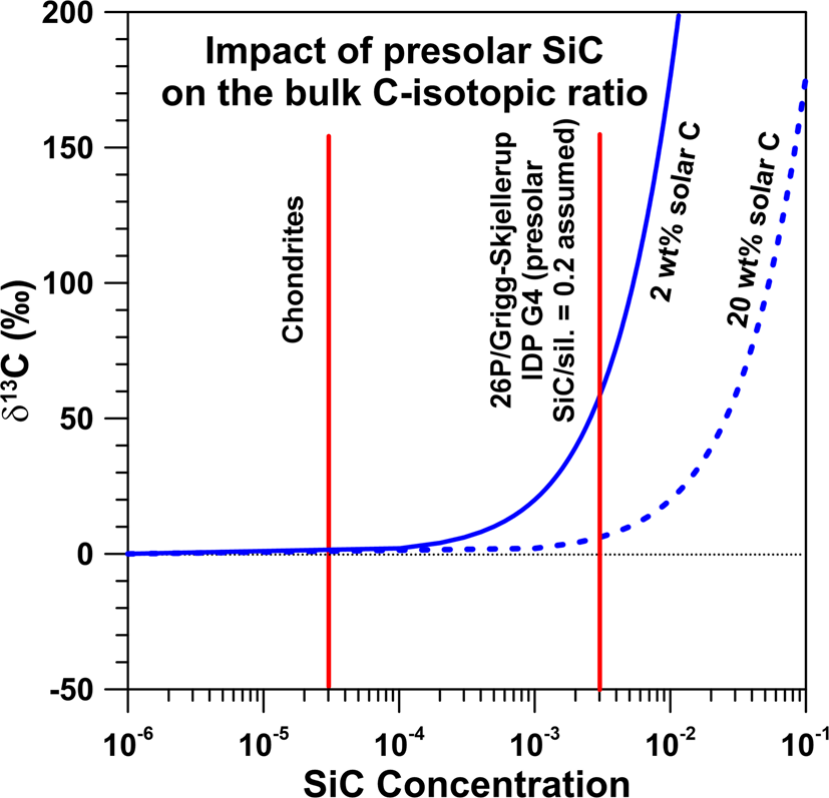
Detectability of presolar SiC grains in matter with 2 wt% (solid blue line) and, respectively, 20 wt% (dashed blue line) of C with terrestrial (solar) C-isotopic composition. Shown are expected $\delta ^{13}$C values as a function of presolar SiC concentration. The left red line represents the SiC concentration in carbonaceous chondrites, the right red line the assumed presolar SiC concentration (0.3%) in an IDP from comet 26P/GS which has the highest O-rich presolar grain abundance (1.5%) among all primitive Solar System materials studied to date. Data sources: presolar SiC concentration in chondrites: Davidson et al. (2014); presolar grain concentration in an IDP from 26P/GS: Busemann et al. (2009)

An important question is whether carbonaceous presolar grains can be identified in 67P/CG based on C-isotopic anomalies. As CO and CO_2_ were released from sublimated ice it is very unlikely that these molecules carry any isotopic signatures of refractory presolar grains. On the other hand, studies of comet 1P/Halley suggested the presence of carbonaceous particles with high ^12^C/^13^C ratios (Jessberger and Kissel 1991) and C-isotopic fingerprints of presolar grains in 67P/CG may be identifiable in dust particles studied by COSIMA. Presolar SiC is the most abundant carbonaceous stardust mineral in primitive meteorites (Zinner 2014). Based on C-, N-, and Si-isotopic compositions it is divided into distinct populations which originate from different types of parent stars (Fig. 3). Figure 4 shows the expected imprint of presolar SiC grains on the bulk C-isotopic composition of the refractory component in 67P/CG as a function of presolar SiC concentration, assuming 2 wt% and, respectively, 20 wt% of C with terrestrial (solar) C-isotopic composition to be present. With the typical abundance (30 ppm) and average C-isotopic composition (enrichment of a factor of ∼2 in ^13^C) of presolar SiC in primitive meteorites, an imprint on the C-isotopic composition would hardly be recognizable; however, with an abundance of 0.3%, as assumed for one IDP associated with comet 26P/GS, which has the highest presolar grain abundance identified in extraterrestrial matter to date (IDP G4; Busemann et al. 2009), the expected anomaly would be 60$\permil$ in $\delta ^{13}$C if the solar C component would account for 2 wt% C. If presolar SiC (or together with other carbonaceous presolar grains with comparable C-isotopic signature) concentrations would be 1%, the anomaly would be as high as 200$\permil$ in $\delta ^{13}$C, which should be recognizable. If the solar C component would account for 20 wt% C, the expected anomalies are smaller. An anomaly with $\delta ^{13}$C of 100$\permil$ would require a presolar SiC (or carbonaceous grains) concentration of a few percent (Fig. 4).

As we discuss below in the context of Si-isotopic compositions, there is a hint for a possible late contribution of matter from a nearby SN to the formation site of 67P/CG. If so, then one could expect to have contributions from carbonaceous SN dust and C-bearing gaseous species, e.g., CO, which forms efficiently in SN ejecta (Cherchneff and Lilly 2008). A fraction of the latter could have been captured by ices that later became building blocks of 67P/CG. Predictions for ^12^C/^13^C in total SN ejecta vary with SN model and can be higher than solar, e.g., ^12^C/^13^C ∼160 in the 15 M_⊙_ SN model of (Rauscher et al. 2002), or lower than solar, e.g., ^12^C/^13^C ∼20 in the model 25T-H10 of Pignatari et al. (2015). The higher contribution of ^13^C in the model 25T-H10 is the result of H ingestion into He shell matter prior to the SN explosion. Qualitatively, the same holds if CO forms more efficiently in zones that are both C- and O-rich. In the context of the Pignatari et al. (2015) model the ^13^C enrichment seen by ROSINA for CO_2_ would be compatible with a late SN contribution. However, as shown below, this does not hold for the O-isotopic composition of CO_2_. If SN grains would dominate not only the O-rich refractory component (see below) but also the C-rich refractory component of 67P/CG then one would expect to find $\delta ^{13}$C values in excess of 100$\permil$ along with large ^15^N enrichments in carbonaceous dust (Fig. 3). COSIMA data will be critical in this respect.

### Oxygen

**Fig. 5:**
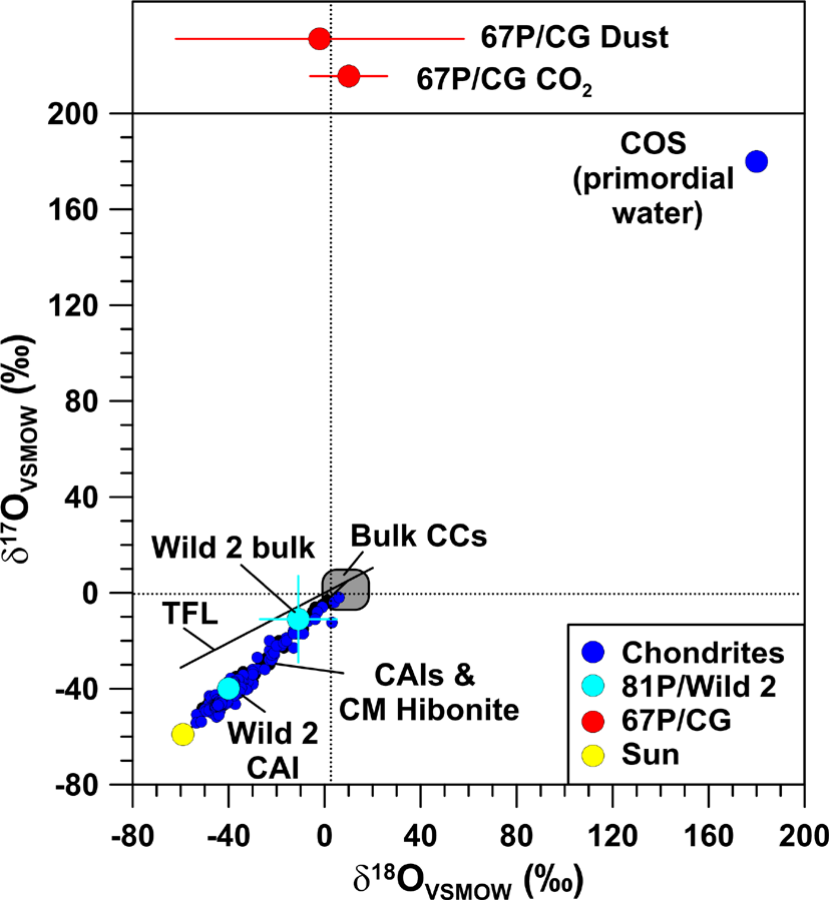
Oxygen-isotopic compositions, given as permil deviation from terrestrial VSMOW, of various components of carbonaceous chondrites, comets 81P/Wild 2 and 67P/CG ($\delta ^{18}$O only, upper panel), and the Sun. Data for Wild 2 bulk composition represent residues in impact craters on Al foils from NASA’s Stardust mission. COS: cosmic symplectite, assumed to represent primordial water in the solar nebula. TFL: Terrestrial fractionation line. Data sources: chondrites: Aléon et al. (2002), Clayton (2004), Fagan et al. (2002), Krot et al. (2002), Liu et al. (2009), Kööp et al. (2016a, 2016b); Lodders and Fegley (1998); Sakamoto et al. (2007); Sun: McKeegan et al. (2011); 81P/Wild 2: McKeegan et al. (2006), Stadermann et al. (2008); 67P/CG: Hässig et al. (2017), Paquette et al. (2018). Errors are 1$\sigma$

**Fig. 6:**
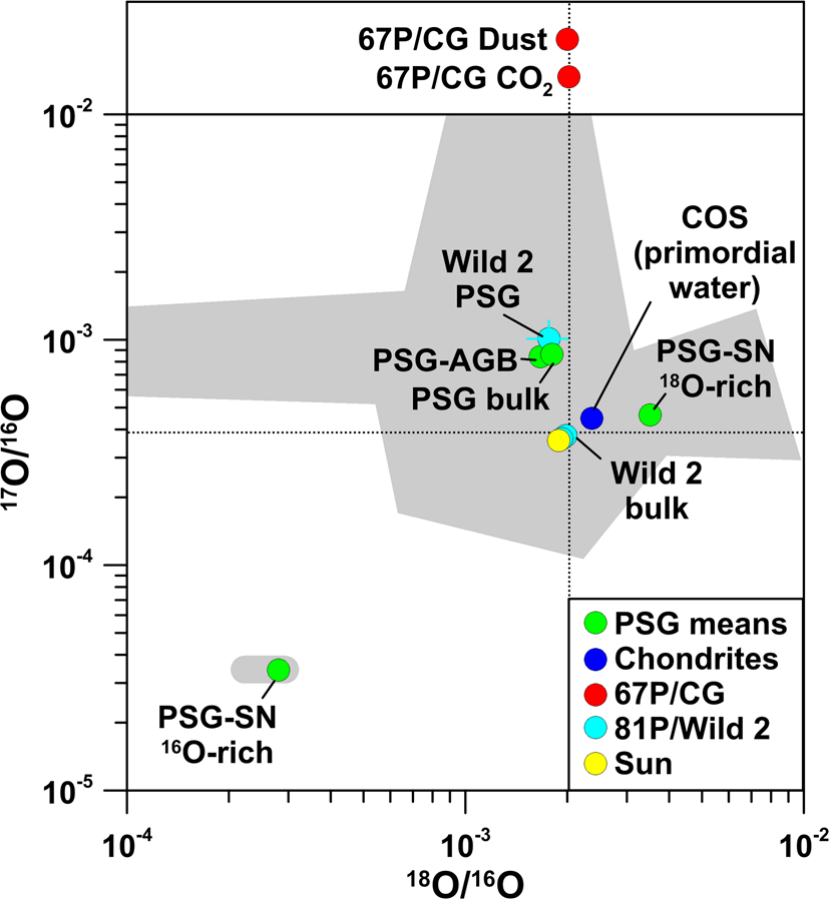
Oxygen-isotopic ratios of specific components in chondrites and comets 81P/Wild 2 and 67P/CG (^18^O/^16^O only, upper panel) in comparison to presolar oxide and silicate grains (individual grains and mean values for grains from AGB stars, supernovae, and novae, and for the bulk of O-rich presolar grains). The light-grey area represents the data of individual presolar silicate and oxide grains. The black dotted lines indicates the terrestrial VSMOW values. PSG: O-rich presolar grains. Wild 2 PSG: a presolar O-rich grain in comet 81P/Wild 2. COS: cosmic symplectite, assumed to represent primordial water in the solar nebula. Data sources: chondrites (COS): Sakamoto et al. (2007); Sun: McKeegan et al. (2011); 81P/Wild 2: McKeegan et al. (2006), Stadermann et al. (2008); 67P/CG: Hässig et al. (2017), Paquette et al. (2018); presolar oxides and silicates: Hynes and Gyngard (2009). Errors are shown for 81P/Wild 2 and 67P/CG only and are 1$\sigma$

There are clear systematics for O in chondrites in that the different groups of chondrites have distinct O-isotopic compositions (Clayton 2004; Lodders and Fegley 1998). Oxygen-isotopic compositions of bulk chondrites are within a few permil of the terrestrial VSMOW standard (Fig. 5); the largest bulk anomalies are seen in CI chondrites which show enrichments in the heavy O isotopes of ∼10$\permil$/amu along the terrestrial mass fractionation line. Much larger O isotope anomalies are evident for CAIs and hibonite grains, which exhibit enrichments in ^16^O of up to 60$\permil$ along a line with slope ∼1 (Aléon et al. 2002; Fagan et al. 2002; Krot et al. 2002; Liu et al. 2009; Kööp et al. 2016a, 2016b) (Fig. 5). The maximum enrichments in ^16^O are compatible with what was inferred for the Sun (McKeegan et al. 2011). Cosmic symplectite (COS), a material consisting of aggregates of nanocrystalline iron sulfide and magnetite, shows large enrichments in ^17^O and ^18^O of about 200$\permil$ (Sakamoto et al. 2007) (Figs. 5 and 6). So far, COS has been unambiguously identified only in the ungrouped carbonaceous chondrite Acfer 094 and is interpreted to have formed by oxidation of Fe, Ni metal and sulfides by primordial ^16^O-poor water in the Solar System. An isotopically similar, but chemically distinct material was recently found in an IDP (Starkey et al. 2014). Another component with extremely large enrichments in ^17^O and ^18^O (^17^O/^16^O and ^18^O/^16^O up to ∼0.1; off-scale in Figs. 5 and 6) are silica-rich grains embedded in organic matter from the Murchison meteorite (Aléon et al. 2005). The preferred interpretation of Aléon et al. (2005) of these anomalies is irradiation of circumsolar gas by high energy particles accelerated during an active phase of the young Sun.

Ground-based spectroscopic observations of several comets have provided ^18^O/^16^O ratios of H_2_O (Bockelée-Morvan et al. 2015, and references therein). Experimental uncertainties (1$\sigma$) are typically ∼10%. All ^18^O/^16^O ratios are compatible with VSMOW within 1.5$\sigma$. For comet 81P/Wild 2 bulk O-isotopic compositions were inferred from studies of impact residues in Al foils (McKeegan et al. 2006; Stadermann et al. 2008). These measurements yielded a small excess in ^16^O of about 10$\permil$, which, however, is fully consistent with VSMOW when the experimental uncertainties of ∼20$\permil$ are considered (Figs. 5 and 6). Recent high-precision studies of 12 impact residues from individual Stardust particles are broadly consistent with this but also revealed two particles slightly enriched or, respectively, depleted in ^16^O by ∼15$\permil$ within uncertainties of about 1$\permil$ (Snead et al. 2017). CAIs were also found in the samples from 81P/Wild 2, which show the typical enrichments in ^16^O as observed in chondrites (McKeegan et al. 2006) (Fig. 5). The observed variations in the ^16^O/^18^O ratio in the ISM range from 200 to 600, and, as in the case of carbon, possibly depend on the distance from the Galactic center (Wilson 1999).

Oxygen-isotopic ratios of the volatile component in comet 67P/CG were measured by ROSINA for H_2_O (Altwegg et al. 2015) and CO_2_ (^18^O/^16^O only; Hässig et al. 2017). The data for H_2_O of Altwegg et al. (2015) are still preliminary and a more detailed study of the oxygen isotopes in water during various stages of the mission is still ongoing. The ^18^O/^16^O ratio of CO_2_ is fully compatible with VSMOW within the experimental uncertainty of ∼20$\permil$ (Hässig et al. 2017) (Fig. 5). Oxygen-isotopic measurements of one dust particle (“Jessica”) in 67P/CG were conducted by COSIMA (Paquette et al. 2018). Data reduction was difficult, because of instrumental artifacts and silicone oil contamination; the inferred ^18^O/^16^O ratio of ($2.00 \pm 0.12) \times 10^{-3}$ is fully compatible with the terrestrial VSMOW value, bulk meteorites, samples from comet 81P/Wild 2 from NASA’s Stardust mission, and the Sun (Fig. 5).

Individual O-rich presolar grains (oxides, silicates) from primitive meteorites show O isotope anomalies that differ significantly from solar. Based on O-isotopic compositions, presolar O-rich grains are divided into 4 distinct groups (Nittler et al. 1997). Most abundant (70–80%) are so-called Group 1 grains which show enhanced ^17^O/^16^O and about solar or slightly lower than solar ^18^O/^16^O. These grains are believed to come from 1.2–2.2 M_⊙_ red giant or AGB stars of about solar metallicity (Nittler 2009). Group 2 grains exhibit strong depletions in ^18^O and enrichments in ^17^O. The proposed stellar sources include low-mass AGB stars that experienced cool bottom processing (Nollett et al. 2003; Wasserburg et al. 1995) and/or intermediate-mass (4–8 M_⊙_) AGB stars (Lugaro et al. 2017). Group 3 grains show slightly enhanced ^16^O and potential stellar sources are AGB stars of low mass and metallicity, and SNe (Nittler et al. 2008). Group 4 grains show enhanced ^18^O/^16^O and a range of ^17^O/^16^O ratios and SNe are the most likely stellar sources (Nittler et al. 2008). Outside this classification scheme are a few grains with very high ^17^O/^16^O ratios of >0.005, which may originate from novae, and two oxide grains with strong ^16^O enrichments from SNe (Gyngard et al. 2010a; Nittler et al. 1998).

**Fig. 7:**
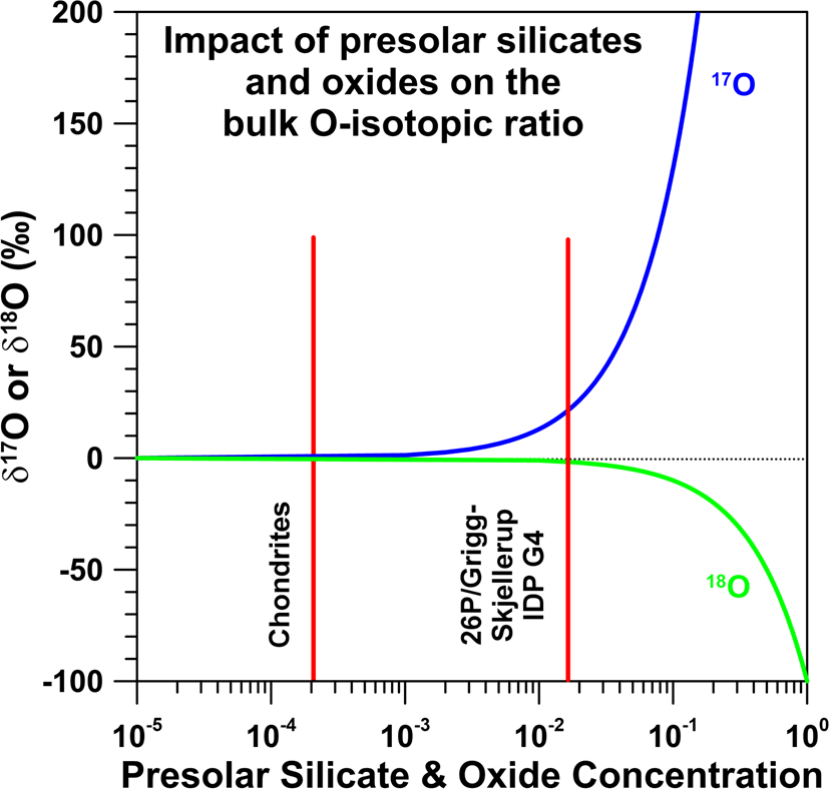
Detectability of presolar silicate and oxide grains in matter of planetary (terrestrial) O-isotopic composition. Shown are expected $\delta$^17,18^O values as a function of presolar silicate and oxide concentration. The left red line represents the concentration of O-rich presolar grains (200 ppm) in the most primitive carbonaceous chondrites, the right red line the concentration of O-rich presolar grains (1.5%) in an IDP from comet 26P/GS which has the highest O-rich presolar grain abundance among all primitive Solar System materials studied to date. In the latter case anomalies in $\delta ^{17}$O of about 20$\permil$ and in $\delta ^{18}$O of a few$\permil$ would be expected. Only concentrations of O-rich presolar grains at the 10 percent level or larger would lead to $\delta ^{17}$O anomalies of ${>} +150\permil$; negative $\delta ^{18}$O anomalies would still be ${<} 100\permil$. Data sources: concentration of O-rich presolar grains in the most primitive chondrites: Floss and Haenecour (2016); Presolar grain concentration in an IDP from 26P/GS: Busemann et al. (2009)

Oxygen-rich presolar grains show on average an enrichment in ^17^O by about a factor of 2 and a depletion in ^18^O by about 10% (Fig. 6). As for carbonaceous presolar grains, O-rich presolar grains are not expected to have left an isotopic fingerprint in the volatile component of 67P/CG, such as H_2_O, CO, and CO_2_. For the refractory part of 67P/CG this might be different. In Fig. 7 we show the expected imprint of O-rich presolar grains as a function of their concentration in matter of otherwise planetary (terrestrial) O-isotopic composition. With the typical concentration (200 ppm) and average O-isotopic composition of O-rich presolar grains in the most primitive chondrites the imprint on O-isotopic ratios would be only a few permil, i.e., not recognizable. For concentrations of presolar grains as observed in IDP G4 (1.5%) the effect would be about 20$\permil$ in $\delta ^{17}$O and even much less in $\delta ^{18}$O. Only if O-rich presolar grains would account for 5–10% of the refractory component of 67P/CG O isotope anomalies in excess of 100$\permil$ in $\delta ^{17}$O can be expected. This concentration of O-rich presolar grains is 3–6× higher than what was observed for IDP G4. If the refractory component in 67P/CG would be dominated by silicates from a SN, as discussed in the context of Si isotopes, large enrichments in ^18^O, the signature of typical presolar SN silicates, would be expected. The first results obtained by COSIMA for the ^18^O/^16^O ratio in the refractory dust component of 67P/CG do not indicate large ^18^O/^16^O anomalies. However, the data are for one particle only and experimental uncertainties are large, so that final conclusions are difficult to draw.

The hypothetical capture of CO from a SN would not only effect the C-isotopic composition but also the O-isotopic composition of the volatile component of 67P/CG. If we take the scenario based on the SN model of Pignatari et al. (2015), as outlined for the C-isotopic ratio, one would expect to find strong depletions in ^18^O along with about solar ^17^O/^16^O in CO from the SN. Depletions of ^18^O in CO_2_ (derived from CO) from ice in 67P/CG would be expected to be of similar magnitude as the predicted enrichments of ^13^C, which is not observed. It thus appears questionable that a late injection of matter from a SN has significantly contributed to the CO inventory of the volatile component in 67P/CG.

### Silicon

Silicon isotope anomalies are generally small (${<}1\permil$) for chondrites on a whole-rock scale (Poitrasson 2017); only for so-called FUN inclusions, a sub-group of CAIs that show large mass-dependent fractionation effects in several elements, Si isotope anomalies as large as 12$\permil$/amu were observed (Clayton et al. 1978). Measurements in the ISM are sparse and in agreement with solar ratios but exhibit considerable error bars (e.g., Tercero et al. 2011; Wilson and Rood 1994).

**Fig. 8:**
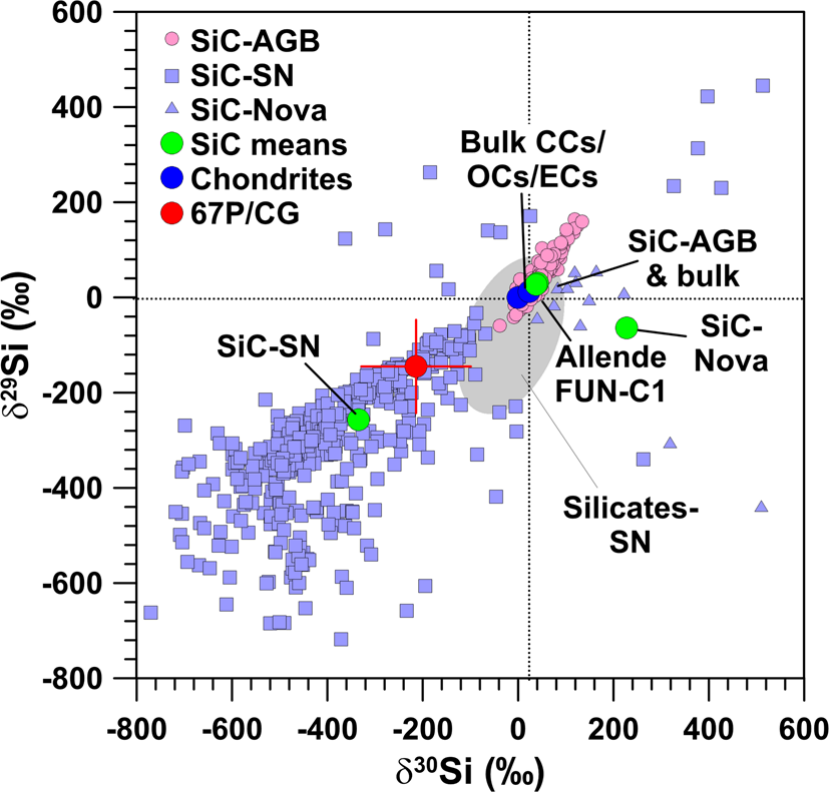
Silicon-isotopic compositions, given as permil deviations from the terrestrial (solar) ratios, of sputtered neutrals from the surface of 67P/CG, specific components in chondrites, presolar SiC grains (individual grains and mean values for grains from AGB stars, supernovae, and novae, and for bulk SiC), and presolar silicates from SNe (grey-shaded area; no individual data points shown). Data sources: 67P/CG: Rubin et al. (2017); chondrites: Clayton et al. (1978), Poitrasson (2017); presolar SiC: Alexander (1993), Amari et al. (2001a), Besmehn and Hoppe (2003); Hoppe et al. (1994, 1996, 2000, 2010, 2012); Huss et al. (1997), Lin et al. (2002), Liu et al. (2016), Marhas et al. (2008), Nittler (1996), Nittler and Alexander (2003), Nittler and Hoppe (2005), Xu et al. (2015); presolar silicates: Floss and Haenecour (2016). Errors are shown for 67P/CG only and are 1$\sigma$

Presolar grains exhibit much larger Si isotope anomalies. Among the Si-rich presolar grains SiC is the best characterized mineral phase. Most of these grains, so-called mainstream grains which account for about 90% of all presolar SiC grains, show enrichments in ^29^Si and ^30^Si of up to 20 percent, plotting along a line with slope 1.37 in a Si three isotope representation (Zinner et al. 2007) (Fig. 8). The SiC mainstream grains formed in the winds of 1–3 M_⊙_ AGB stars of about solar metallicity (Lugaro et al. 2003) and the slope 1.37 line is interpreted to represent the Galactic chemical evolution, both in time and space, of the Si isotopes. Much larger Si isotope anomalies are evident for SiC grains from SNe, the X and C grains, which make up 1–2% of all presolar SiC grains (Zinner 2014). X grains show strong depletions in the heavy Si isotopes with average $\delta$^29,30^Si values of about −300$\permil$ (Fig. 8); C grains are about 10 times less abundant than X grains and show large enrichments in the heavy Si isotopes of up to a factor of >4. Presolar Si_3_N_4_ grains are very rare, comparably to the SiC C grains, and share the Si-isotopic signature of X grains. Presolar silicates are about 5× more abundant than presolar SiC grains (Floss and Haenecour 2016); however, because they cannot be separated chemically from meteorites, their Si isotope compositions have to be measured in situ in meteoritic thin sections and given their small size of typically 100–400 nm, dilution with Si from surrounding material of Solar System origin may have severely compromised the existing Si isotope data of presolar silicates, and true isotope anomalies might be more extreme (Nguyen et al. 2007). Group 1 presolar silicates, which originate from red giant and AGB stars, dominate the presolar silicate inventory and these grains show Si isotope compositions often compatible with the terrestrial (meteoritic) values, albeit within rather large experimental uncertainties of several percent (Floss and Haenecour 2016). Presolar silicates with a likely SN origin, the Group 4 and some of the Group 3 grains, constitute about 10–20% of presolar silicates (Floss and Haenecour 2016). Only one of these putative SN silicates has the typical Si-isotopic signature of SiC X grains (Messenger et al. 2005) (Fig. 8); for some other SN silicates smaller depletions in the heavy Si isotopes are evident as well, but many grains with a putative SN origin also show close-to-solar Si-isotopic ratios (Floss and Haenecour 2016) (Fig. 8).

**Fig. 9:**
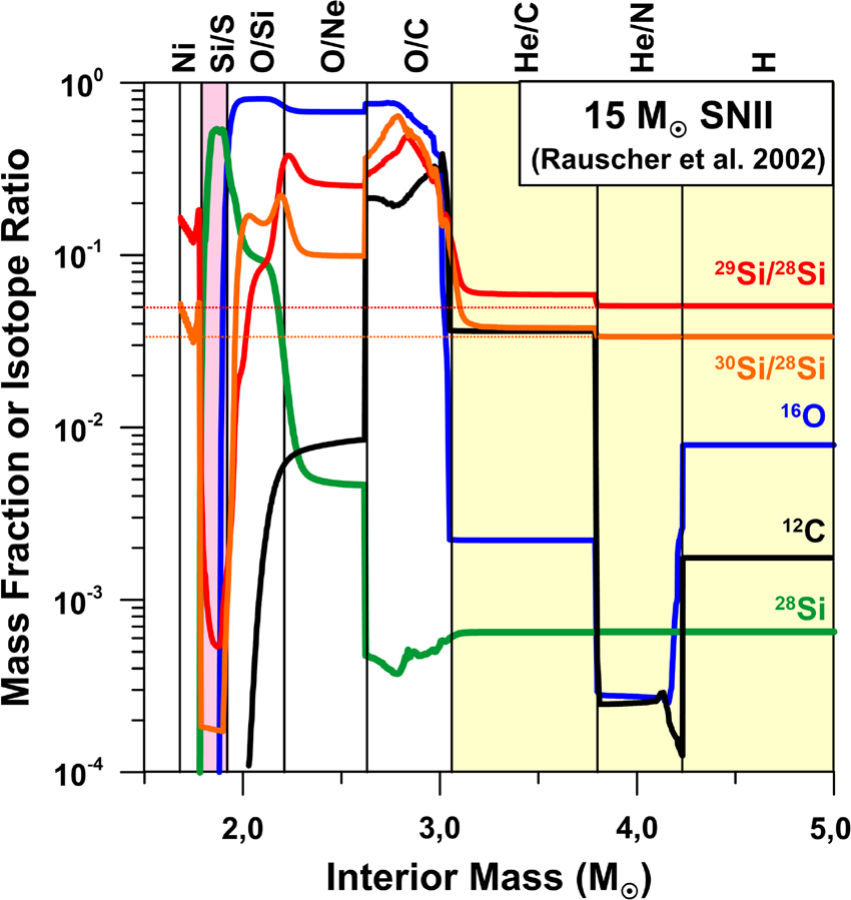
Profiles of mass fractions of C, O, and Si, and of Si-isotopic ratios in the interior of a 15 M_⊙_ Type II SN according to Rauscher et al. (2002). The names of SN zones are indicated at the top of the figure and follow the nomenclature of Meyer et al. (1995). The terrestrial (solar) Si-isotopic ratios are indicated by horizontal dotted lines. The pink colored area denotes the mass region in which Si is very abundant and exhibits strong depletions in the heavy Si isotopes (Si/S zone). The yellow colored area denotes zones (He/C, He/N, H) that must have contributed to presolar silicate grains as to account for their O-isotopic signatures. Admixture of matter from the Si/S zone to these outer zones could easily produce silicates with isotopically light Si (while leaving O-isotopic compositions largely unaffected). The total ejecta shows a depletion in ^29^Si ($\delta ^{29}\mathrm{Si} = -400\permil$) and an enrichment in ^30^Si ($\delta ^{30}\mathrm{Si} = 300\permil$)

The Si-isotopic composition of the refractory component of 67P/CG was measured by ROSINA from sputtered Si neutrals, produced by bombardment of the cometary surface by solar wind protons (Rubin et al. 2017; Wurz et al. 2015). Moderate depletions of 10–20% of the heavy Si isotopes were observed, with a significance at the 1–2$\sigma$ level. As discussed by Rubin et al. (2017), part of these depletions might be caused by an unrecognized fractionation during sputtering with solar wind protons. Nevertheless, it is also well conceivable that the observed depletion in the heavy Si isotopes is real, which in view of the only small Si isotope variations in materials of Solar System origin has interesting and important implications. As we have discussed above, isotopically light Si is found in presolar SiC and Si_3_N_4_ grains from SNe; for silicates from SNe this is less clear, but given that Si isotope anomalies might be more extreme than observed, because of dilution effects, a depletion of the heavy Si isotopes of a few percent on average cannot be excluded. Negative Si isotope anomalies are easily achieved in SN models. In the context of the 15 M_⊙_ SN model of Rauscher et al. (2002) the observed O isotope characteristics of Group 4 silicates are well explained by contributions from the outer He/C, He/N, and H zones (Fig. 9). Admixture of matter from the interior Si/S zone, in which Si is essentially monoisotopic ^28^Si (Fig. 9), will lead to lower-than-solar ^29^Si/^28^Si and ^30^Si/^28^Si ratios, as observed in 67P/CG. This selective mixing approach is not without problems but was quite successful in matching the isotope ratios of many elements in SiC X grains (e.g., Hoppe et al. 2010). The model of Pignatari et al. (2015) avoids this selective, large-scale mixing approach in the context of presolar SiC grains, as it predicts a C- and Si-rich (C/Si) zone at the bottom of the He-burning shell, which, with admixture of matter from thin layers below and above, can account for the isotopic compositions of SiC SN grains (Hoppe et al. 2018). The isotopic compositions of O-rich presolar grains have not yet been explored in the context of the Pignatari et al. (2015) models. We note, however, that below the C/Si zone there is an O- and Si-rich zone which shows strong depletions in the heavy Si isotopes. Oxygen in this zone is essentially monoisotopic ^16^O, a signature which is rarely observed in presolar SN grains. It remains to be seen whether the SN models of Pignatari et al. (2015) can be modified as to account for the isotopic signatures of ^18^O-rich SN silicates without considering selective, large-scale mixing.

In any case, if we want to account for depletions in the heavy Si isotopes in the refractory component of 67P/CG we must assume that this component is dominated by dust from a SN. This could be a hint for a local, late contribution of a nearby SN to the solar nebula. It is interesting to note in this context that among the seven presolar silicates identified in IDP G4, associated with comet 26P/GS, four belong to the rare Group 3 (Busemann et al. 2009); the origin of grains from this group is still a matter of debate and SNe are among the proposed stellar sources. Moreover, the only likely presolar SiC grain identified in IDP G4 is an X grain, i.e., has a SN origin, which is remarkable given the fact that X grains contribute only 1–2% to the presolar SiC inventory in chondrites. Similarly, Yada et al. (2008) observed a clustering of SN Group 4 silicates in Antarctic micrometeorites. These observations suggest that different types of presolar grains might have been heterogeneously distributed among different Solar System bodies.

### Sulfur

**Fig. 10:**
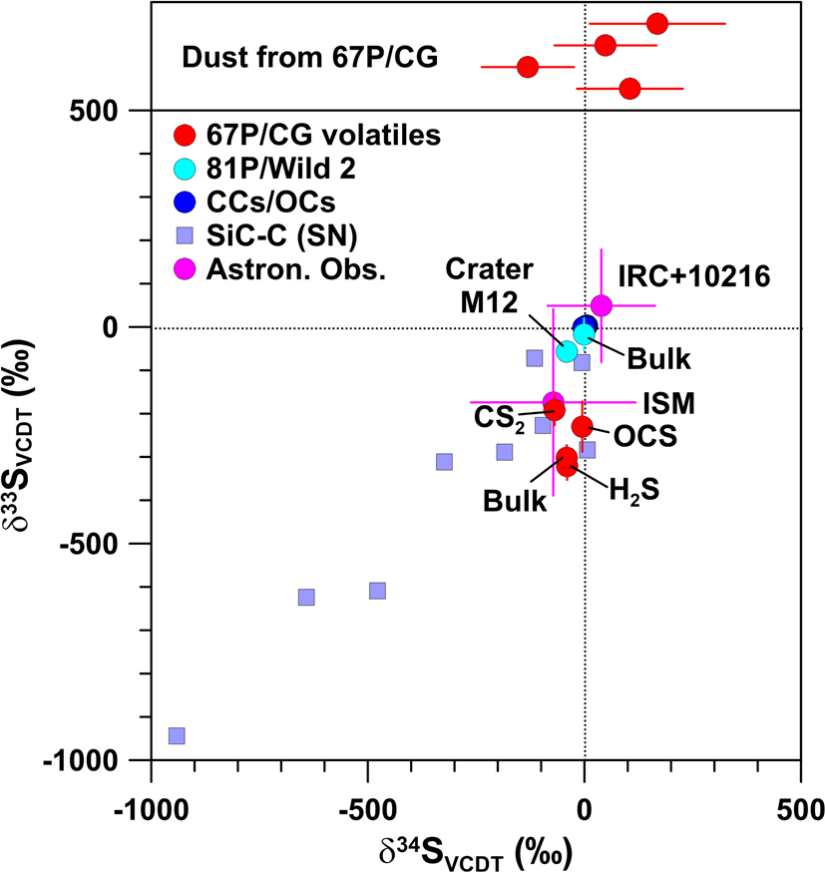
Sulfur-isotopic compositions, given as permil deviation from the VCDT standard, of bulk carbonaceous and ordinary chondrites (CCs/OCs), residues in impact craters on Al foils from NASA’s Stardust mission to comet 81P/Wild 2, different molecules (as well as their weighted mean; H_2_S and CS_2_ data from October 2014, OCS data from May 2016) from comet 67P/CG measured by ROSINA, and four dust grains from comet 67P/CG studied by COSIMA (upper panel, ^34^S/^32^S data only) in comparison to presolar SiC Type C grains from SN explosions, astronomical observations of carbon star $\mathrm{IRC}+10216$, and the ISM. Data sources: chondrites: Bullock et al. (2010), Gao and Thiemens (1993a, 1993b); 81P/Wild 2: Heck et al. (2012); 67P/CG: Calmonte et al. (2017), Paquette et al. (2017); presolar SiC: Gyngard et al. (2010b); Hoppe et al. (2012, 2018); Liu et al. (2016), Xu et al. (2015); ISM and $\mathrm{IRC}+10216$: Mauersberger et al. (2004). Errors are shown for 81P/Wild 2, 67P/CG, the ISM, and $\mathrm{IRC}+10216$ only and are 1$\sigma$

**Fig. 11:**
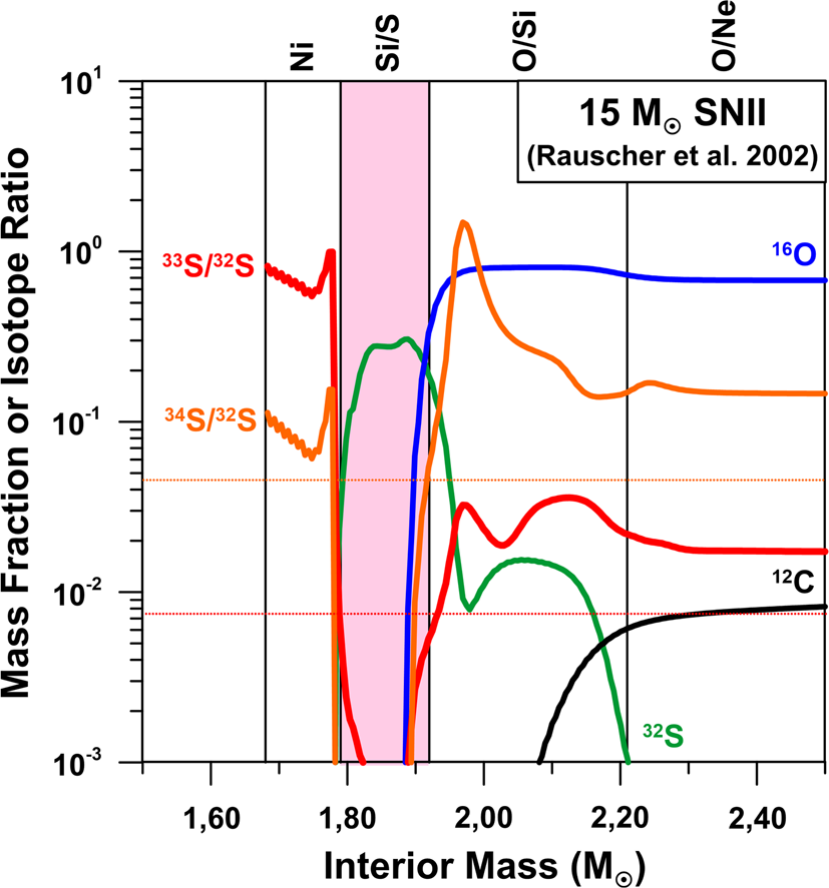
Profiles of mass fractions of C, O, and S, and of S-isotopic ratios in the interior of a 15 M_⊙_ Type II SN according to Rauscher et al. (2002). The names of SN zones are indicated at the top of the figure and follow the nomenclature of Meyer et al. (1995). The terrestrial (solar) S-isotopic ratios are indicated by horizontal dotted lines. The pink colored area denotes the mass region in which S is very abundant (Si/S zone), providing favorable conditions for the formation of S-bearing molecules and dust in the ejecta after explosion. Sulfur is isotopically light in this region. The total ejecta shows a moderate depletion in ^33^S ($\delta ^{33}\mathrm{S} = -390\permil$) and a small enrichment in ^34^S ($\delta ^{34}\mathrm{S} = 120\permil$)

Sulfur isotope anomalies are generally small (a few permil) in chondrites on a whole-rock scale (Bullock et al. 2010; Gao and Thiemens 1993a, 1993b). Individual presolar SiC grains from AGB stars have ^33^S/^32^S and ^34^S/^32^S ratios compatible with the terrestrial VCDT standard within the experimental uncertainties of several percent; means are $\delta ^{33}\mathrm{S} = -8 \pm 56\permil$ and $\delta ^{34}\mathrm{S} = 13 \pm 23\permil$ (Hoppe et al. 2015). In contrast, large S isotope anomalies are observed in presolar SiC grains from SNe, especially in C grains (Gyngard et al. 2010b; Hoppe et al. 2012, 2018; Liu et al. 2016; Xu et al. 2015). These grains show large excesses in ^32^S of up to a factor of 20 relative to terrestrial S (Fig. 10). The preferred interpretation of these excesses is decay of radioactive ^32^Si (half life 153 yr), which is predicted to be produced in SNe in significant amounts and which condenses along with the stable Si isotopes into SiC (Pignatari et al. 2013). An alternative explanation is preferred incorporation of isotopically light S from the inner Si/S zone in SNe (Fig. 11) due to S molecule chemistry in SN ejecta (Hoppe et al. 2012).

Prior to Rosetta, the ^34^S/^32^S ratio was measured in molecular CS and H_2_, and as S^+^ ions in the coma of three comets (Bockelée-Morvan et al. 2015, and references therein). All ratios are compatible with the terrestrial VCDT standard within the experimental 2$\sigma$ uncertainties of 20–50%.

Sulfur isotope compositions in the volatile component of 67P/CG were measured by ROSINA for several molecules, namely, H_2_S, CS_2_, and OCS (Calmonte et al. 2017). These measurements yielded large depletions in ^33^S along with smaller depletions in ^34^S in all molecules, with mass-weighted means of $\delta ^{33}\mathrm{S} = -302 \pm 29\permil$ and $\delta ^{34}\mathrm{S} = -41 \pm 17\permil$ (Fig. 10). This is clearly different from S on the Earth, in meteorites, in comet 81P/Wild 2 (Heck et al. 2012), and in the atmosphere of carbon star IRC+10217 (Mauersberger et al. 2004), but compatible with S in the ISM (Mauersberger et al. 2004), albeit the latter has large experimental 1$\sigma$ uncertainties of ∼20% (Fig. 10). Measurements of the ^34^S/^32^S ratio on four dust particles by COSIMA gave values compatible with the terrestrial ratio, albeit within large uncertainties, and a mean $\delta ^{34}$S of ${+}48 \pm 129\permil$ (Paquette et al. 2017).

An interesting question is whether the S isotope anomalies in the volatile component of 67P/CG could be an imprint of a late SN contribution, as discussed above. As ^32^Si is likely to be incorporated into Si-rich dust, it is not expected that its decay to ^32^S has left an imprint in the volatile component of 67P/CG. On the other hand, SNe are expected to form substantial amounts of S-bearing molecules in the ejecta. The inner regions of SN ejecta show high abundances of isotopically light S (Fig. 11) and formation of SiS molecules and FeS molecule clusters is predicted (Cherchneff and Dwek 2009, 2010). Iron sulfide may even condense into dust in the inner zones of unmixed SN ejecta (Cherchneff and Dwek 2010), but whether it survives extended time periods after its production in the harsh SN environment remains questionable. Molecular SO is expected to form in the intermediate O-rich SN zones (Cherchneff and Dwek 2009, 2010), in which S is isotopically heavy (Fig. 11). Molecular and atomic S will be expelled after the explosion and might have been trapped by interstellar ices that later became part of 67P/CG. Transformation of SN-derived S to molecules as observed in 67P/CG could have occurred in the ice or during sublimation of ice. The expected S-isotopic fingerprint would be that of the total SN ejecta. In the context of the 15 M_⊙_ SN model of Rauscher et al. (2002) the total ejecta has $\delta ^{33}\mathrm{S} = -390 \permil$ and $\delta ^{34}\mathrm{S} = +120\permil$. This is remarkably close to what was observed in 67P/CG. Whether a SN contribution would be recognizable in the S-isotopic pattern of 67P/CG depends critically on the ratio of SN-derived S and S that was already present in the ices that later became part of 67P/CG and requires that the latter was present at distinctly lower levels than the S from a late SN contribution.

### Argon and Xenon

**Fig. 12:**
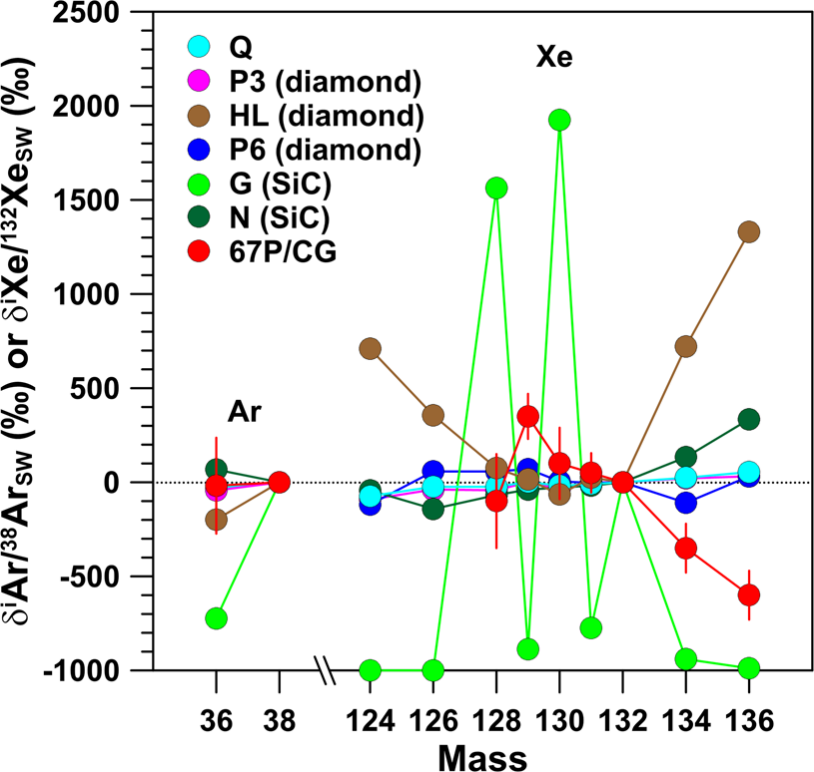
Argon- and Xe-isotopic compositions, given as permil deviation from solar wind Ar and Xe, respectively, of different components in meteorites and of Ar and Xe released from sublimation of ice in 67P/CG. Components P3, HL, and P6 are contained in presolar diamonds, G and N in presolar SiC. Data sources: Balsiger et al. (2015), Marty et al. (2017), Ott (2014). Errors are shown for 67P/CG only and are 1$\sigma$

Meteorites contain trapped noble gases and those produced in situ by spallation or radioactive decay. For a detailed review see Ott (2014). The first category includes implanted solar wind noble gases and so-called ‘planetary gases’, which were trapped early in the history of the Solar System. The trapped planetary noble gases exhibit strong elemental fractionations; relative to solar wind the heavy noble gases are strongly enriched relative to the light ones. The trapped planetary noble gases can be divided into various components (Q, P3, HL, P6, G, N), which exhibit distinct isotopic patterns. Except Q, all components are considered presolar. The Q component dominates the heavy (Ar, Kr, Xe) noble gas inventory of primitive meteorites and its isotopic compositions are similar to those of the solar wind (Fig. 12). The largest isotope anomalies are seen in the HL component, which is contained in presolar diamonds, and in the G component, which is contained in presolar SiC and which represents the isotopic signature of the s-process in AGB stars (Fig. 12).

For 67P/CG isotopic compositions of Ar and Xe, released by sublimation of ice, were measured by ROSINA. The ^36^Ar/^38^Ar ratio, determined as $5.4\pm 1.4$ (Balsiger et al. 2015), is compatible with that of the Earth, solar wind, and all trapped planetary components, except the G component (Fig. 12). The most pronounced signature of Xe are depletions in the heavy isotopes ^134^Xe and ^136^Xe relative to ^132^Xe of 35% and 60%, respectively (Marty et al. 2017) (Fig. 12). The Xe-isotopic pattern of 67P/CG does not match any of the patterns of the trapped planetary noble gas components, neither for the Q component nor for the components associated with presolar grains (Fig. 12). However, as discussed by Marty et al. (2017), the Xe pattern of 67P/CG can be matched relatively well by a mixture of s-process Xe and two r-process endmember compositions identified by Gilmour and Turner (2007). By mixing 22% of cometary Xe as in 67P/CG with Q–Xe it is possible to match the pattern of the theoretically defined primordial atmospheric U–Xe component on Earth. This provides evidence that comets contributed to atmospheric Xe on Earth, or more generally to atmospheric noble gases (Marty et al. 2017).

Like for the other volatiles discussed above, we can compare the Ar- and Xe-isotopic compositions predicted for the total ejecta of a single SN with those of 67P/CG. If we consider the 15 M_⊙_ and 25 M_⊙_ SNII models of Rauscher et al. (2002), predicted Ar- and Xe-isotopic patterns, except for the ^36^Ar/^38^Ar ratio in the 25 M_⊙_ SN model, differ significantly from what is observed in 67P/CG; in particular the observed depletions in ^134^Xe and ^136^Xe cannot be reproduced. This suggests that the Xe was already present in the ices that later formed comet 67P/CG and was not delivered by a hypothetical nearby SN.

## Summary and Outlook

ESA’s Rosetta mission to comet 67P/CG has provided a wealth of isotope data that allow to get new insights into the origin of the Solar System. As of June 2018, isotopic compositions of H, C, O, S, Ar, and Xe were determined by ROSINA for the volatile component of 67P/CG, released by sublimation of ice. Complementary isotope data were obtained for Si in the refractory component of 67P/CG, determined by ROSINA from sputtered Si neutrals, produced by bombardment of the cometary surface by solar wind protons, and for O and S from several dust particles analyzed by COSIMA. The isotopic compositions of 67P/CG show similarities and differences with other primitive Solar System materials, namely, primitive meteorites, IDPs, UCAMMs, matter from comet 81P/Wild 2 returned by NASA’s Stardust mission, and several other comets studied from space and Earth:

1. Water in 67P/CG has a high D/H ratio of 3.4× the terrestrial VSMOW standard that falls at the upper end of what is observed for comets and which is distinctly higher than the D/H ratio of water in carbonaceous chondrites. D/H is even higher in the doubly deuterated water, D_2_O. This suggests that comet 67P/CG might be particularly primitive and might have preserved large amounts of presolar matter, including refractory presolar grains.
2. CO_2_ in 67P/CG exhibits small enrichments in ^13^C of ∼6% relative to the terrestrial PDB standard. Data for CO are compatible with this, albeit experimental uncertainties are large. The ^13^C/^12^C ratio of CO_2_ is compatible with that of carbonates in meteorites but slightly higher (with 1–2$\sigma$ significance) than the ratios of bulk carbonaceous chondrites and comet 81P/Wild 2. Carbonaceous presolar grains, on the other hand, exhibit much larger C isotope anomalies than the volatile component of 67P/CG, with a mean enrichment of ^13^C of about a factor of 2 in presolar SiC.
3. Within experimental 2$\sigma$ uncertainties, the O-isotopic ratios of CO_2_ in 67P/CG agree with those of bulk chondrites and comet 81P/Wild 2. Presolar silicates and oxides show much larger O-isotopic anomalies than the volatile component of 67P/CG, with a mean enrichment of ^17^O of about a factor of 2 and a depletion of 10% in ^18^O.
4. Refractory Si in 67P/CG shows a depletion in the heavy Si isotopes with 1–2$\sigma$ significance relative to terrestrial and meteoritic Si-isotopic composition. Low ^29^Si/^28^Si and ^30^Si/^28^Si ratios are the signature of presolar SN grains, suggestive of a possible late significant contribution of Si-rich dust from a nearby SN to the formation site of 67P/CG.
5. Sulfur-isotopic compositions of H_2_S, CS_2_, and OCS in 67P/CG show large anomalies relative to terrestrial and meteoritic S with a significance of more than 10$\sigma$ for the mean ^33^S/^32^S. The depletions of ∼30% in ^33^S and of ∼4% in ^34^S are compatible with S in the ISM, albeit within the large experimental uncertainties of the latter. Depletions in the heavy S isotopes are also evident for presolar SiC grains of Type C, believed to come from SNe, probably the imprint of radioactive ^32^Si (half-life 153 yr) decay. Decay of ^32^Si is not expected to have contributed significantly to ^32^S in the volatile component of 67P/CG.
6. While the ^36^Ar/^38^Ar ratio in 67P/CG is compatible with solar wind Ar, Xe in 67P/CG deviates significantly from solar wind Xe and from trapped noble gas components in chondrites. The most pronounced signature of Xe are depletions in the heavy isotopes ^134^Xe and ^136^Xe. The observed isotope pattern can be matched relatively well by a mixture of s-process Xe and two r-process endmember Xe compositions.
7. Refractory presolar grains are not expected to have left isotopic fingerprints in the volatile component of 67P/CG. However, as suggested by the Si data, we have explored the expected fingerprints from a contribution from a nearby SN on the isotopic compositions of the volatiles in 67P/CG. While the S-isotopic compositions of volatile species are consistent with predictions for a 15 M_⊙_ SN, the isotope data of C and O of CO_2_ and of Xe are inconsistent with a contribution from a single SN.

The reduction of isotope data from 67P/CG is still in progress and much more information can be expected in the years to come. Of particular importance will be H-, C-, and N-isotopic data of specific molecules from organics in the volatile component of 67P/CG. A detailed study of ^18^O/^16^O in water from 67P/CG is still ongoing and it will be interesting to see how it compares with meteoritic COS, which has enrichments in ^17^O and ^18^O of about 20%, and which was assumed to represent the O-isotopic signature of primordial water in the solar nebula. Carbon- and O-isotopic data from COSIMA for carbonaceous and O-rich dust are critical to estimate the fraction of presolar grains (stardust) in the refractory component of 67P/CG. Presolar grain abundances at the percent level would lead to C- and O-isotopic anomalies of 10% or larger at bulk scales. These data could also help to constrain the hypothetical contribution from a nearby SN to comet 67P/CG because, as inferred from presolar grains in meteorites and IDPs, one would expect to find the specific isotopic fingerprints of presolar SN grains.
